# Developing a serious game for gaze stability rehabilitation in children with vestibular hypofunction

**DOI:** 10.1186/s12984-023-01249-x

**Published:** 2023-09-26

**Authors:** José Ortega Solís, Pierre Reynard, Karen Spruyt, Cécile Bécaud, Eugen Ionescu, Hung Thai-Van

**Affiliations:** 1 Service d’Audiologie & Explorations Oto-Neurologiques, Hospices Civils de Lyon, 5 Place d’Arsonval, Lyon, 69003 France; 2 Present Address: Société française de kinésithérapie vestibulaire, Toulouse, France; 3 Université Claude Bernard Lyon 1, Lyon, France; 4 Centre de Recherche et d’Innovation en Audiologie Humaine, Institut Pasteur, Institut de l’Audition, rue du Docteur Roux, Paris, 75015 France; 5 Université Paris Cité, NeuroDiderot - INSERM, Hôpital Robert Debré AP-HP, 48 Bd Sérurier, Bingen, Paris, 75019 France

**Keywords:** Serious game, Children, Physiotherapy, Vestibular rehabilitation, Vestibular hypofunction, Gaze stability exercises

## Abstract

**Background:**

Children with vestibular hypofunction (VH) may have gaze instability, balance disorders, and delayed postural-motor development. Gaze stabilization exercises (GSE) are designed to improve dynamic visual acuity (DVA). We aimed to assess the acceptability of a serious game prototype called Kid Gaze Rehab (KGR) designed to implement GSE training in children with VH, combined with traditional vestibular rehabilitation. Effects on DVA and motor performance were also analyzed.

**Methods:**

Twelve children (6 to 9 years old) were included. Sessions were held at the hospital twice a week, for 5 weeks. An adapted French version of The Child Simulator Sickness Questionnaire (SSQ) and the Face Scale Pain-Revised (FPS-R) were used to assess pain in the cervical region and undesirable side effects after each session. Vestibular and motor function parameters (active and passive DVA and Movement Assessment Battery for Children–Second Edition, MABC-2) were assessed before and after the training.

**Results:**

All children included completed the 10 sessions. The FPS-R visual analog scale and SSQ showed good cervical tolerance and no oculomotor or vegetative adverse effects nor spatial disorientation. After training, active DVA scores were significantly improved for the right, left, and up directions (p < 0.05). Passive DVA scores were significantly improved for the left and down directions (p < 0.01 and p < 0.05, respectively). MABC-2 scores were improved in the balance and ball skill sections (p < 0.05).

**Conclusion:**

An innovative pediatric training method, the use of a dedicated serious game for gaze stabilization was well-tolerated as a complement to conventional vestibular rehabilitation in children with VH. Moreover, both DVA and motor performance were found to improve in the study sample. Although replication studies are still needed, serious game-based training in children with VH could represent a promising rehabilitation approach for years to come.

**Trial registration:**

The study was conducted in accordance with the Declaration of Helsinki and approved by an Institutional Review Board (local ethics committee, CPP Sud-Est IV, ID 2013–799). The study protocol was registered on ClinicalTrials.gov (NCT04353115).

**Supplementary Information:**

The online version contains supplementary material available at 10.1186/s12984-023-01249-x.

## Background

Vestibular dysfunction is a disturbance in the vestibular system due to a pathology affecting the peripheral or central vestibular system [1]. The prevalence of vestibular dysfunction in the general pediatric population has yet to be determined. Scattered studies have indicated a rate between 0.45 and 15% [2, 3]. Alternatively, vestibular dysfunction prevalence resulting from hypofunction has been reported to be high in children with sensorineural hearing loss (SNHL), in whom vestibular hypofunction (VH) ranges from 20 to 85% [4, 5]. Given the close anatomical relationship and the common embryological origin of the cochlea and vestibulum, it is not surprising that SNHL children may exhibit some degree of vestibular dysfunction [5]. Children with VH can have gaze instability during head movements, balance disorders, and delayed postural-motor development [6]. Unlike adults with VH, vertigo is not typically reported, except for the acute unilateral loss of vestibular function [7]. For young children, vertigo and dizziness are also more difficult to verbalize [8]. Thus, the most apparent manifestation of VH in children is often balance disorders [4, 9]. A deficit of the vestibular-ocular reflex (VOR) is another consequence of losing vestibular function. VOR stabilizes the image in the retina during head movements by producing eye movements in the opposite direction to head rotation [10]. A VOR impairment may cause a delay in eye movement during head rotations, leading to retinal image slip. Even small amounts of retinal image slip such as 3°/s can significantly decrease visual acuity [11]. Dynamic visual acuity (DVA) is the capacity to fixate on a stable target and see it clearly during head movements [10]. It is commonly measured as the difference between visual acuity when the head is stationary and when it is moving [10]. The VOR deficiency may cause blurred vision, oscillopsia, or a loss of DVA when doing fast head movements above 100°/s [6, 12]. Besides the perturbation of vision during head movements, VOR deficiency can impact reading ability and, therefore, school performance [13].

Vestibular physiotherapy, or vestibular rehabilitation (VR), is a safe and efficient intervention for patients with uncompensated VH [14, 15]. The main exercises used in VR are divided into three groups: gaze stabilization exercises (GSE), habituation exercises, and balance training [14, 15]. Moderate to strong evidence supports VR in managing adults with unilateral and bilateral VH [14]. Although there is a paucity of research on VR in children, results from studies suggest that children with VH respond to VR analogously to adults [6, 16, 17]. GSE are designed to improve the capacity of the eyes to reach or stay on a target during head movements [10, 14]. In the most used paradigm, the patient visually fixates on a stationary or dynamic target and rotates the head in either the yaw or pitch plane. GSE training can improve DVA in adults with VH by raising the VOR gain and/or inducing changes in compensatory saccades [10]. In a controlled case study measuring the efficacy of GSE training in children with bilateral VH, Braswell and Rine reported improvements in the DVA and reading capacity [18]. However, clinical experience shows that GSE is more difficult to implement with children than other rehabilitation exercises like balance training [16]. Indeed, active GSE may be challenging for young children to understand; they may be perceived as dull or constraining tasks, thus impacting training compliance.

Technological advances have promoted new therapeutic methods such as virtual reality and serious games. Research shows serious games are a safe, pleasant, and motivating option in the field of pediatric physical therapy rehabilitation [19]. Regarding its application in VR and, more precisely, in GSE training, Chen et al. have used an interactive video game designed for GSE training in adult patients with VH [20]. The system allowed head motion detection in real-time and set an angular acceleration threshold to increase the exercise’s difficulty level gradually. Balance and DVA improvements were recorded in all participants. Micarelli et al. completed a randomized trial study combining a classical VR protocol with a head-mounted virtual reality device [21]. Adult patients with unilateral VH were assigned either to the VR group or to the VR and virtual reality training group. The virtual reality protocol included balance training, habituation, and GSE. Postural stability, VOR gain, and balance confidence improved in the VR and virtual reality training group.

The primary purpose of this study was to assess the acceptability and feasibility of a serious game prototype called Kid Gaze Rehab (KGR), which was specifically designed to implement GSE training in children with SHL and unilateral or bilateral VH. The effects of KGR on VOR function as well as motor performance were also investigated.

## Methods

### Participants and data collection

Twelve children with SNHL followed in our audiology and otolaryngology department were included (mean age = 6 years and 2 months; range 5 years and 6 months to 9 years and 6 months) (Table 1). The inclusion criteria were children from 4 to 13 years old with peripheral unilateral or bilateral VH, participants able to understand the terms and purpose of the study, and the non-opposition of children and parents. The exclusion criteria were cervical spine disorder and vision impairment (uncorrected refractive error or eye movement disorders other than VOR impairment). The local ethics committee approved the study protocol; the study project has been registered on ClinicalTrials.gov (NCT04353115). Written informed consent and assent were obtained.

Demographic and clinical data collected were as follows: age, sex, hearing status, cervical vestibular evoked myogenic potentials (cVEMPs), and VOR gain using the Video Head Impulse Test (VHIT). The cVEMPs were obtained in bone conduction (Neuro Audio system, Collin®, France); the response was rated present (+) if a reproducible wave P13-N23 of at least 50uV in amplitude was present; if not, it was rated as absent (-) (Table 1).

**Table 1:** Demographic and clinical data of included children. RE: Right ear; LE: Left ear, cVEMP: cervical vestibular evoked myogenic potentials; IC: cochlear implant, 2 when bilateral; MHL, PHL: moderate and profound hearing loss; VOR: vestibulo ocular reflex; AC, LC, PC: anterior, lateral, and posterior canal

Child	Age	Sex	Hearing status	cVEMPs RE/LE	VOR gain at inclusion AC/LC/PC	
					Right ear	Left ear
**1**	8y	M	IC2	**+/-**	1/1/0.8	0.7/0.4/0.4
**2**	6y 10 m	F	PHL (LE)	**-/-**	0.9/0/0	0.2/0/0
**3**	8y 10 m	M	SHL (LE)	**+/+**	0.6/0.7/0.4	0.1/0/0.3
**4**	5y 6 m	M	IC2	+/+	0.6/0.3/0.1	0.8/1/0.2
**5**	5y 11 m	M	MHL2	**+/-**	0.2/0.9/0	0.6/0.6/0
**6**	5y 4 m	M	IC2	**-/-**	0.4/0/0	0.4/0/0
**7**	7y 8 m	M	IC2	**-/-**	0.7/0.6/0.1	0.7/0.5/0
**8**	6y 2 m	F	PHL (RE); MHL (LE)	**+/-**	0.3/0/0	0/0/0
**9**	7y 3 m	M	PHL (LE)	**+/-**	1/1/0.7	0.2/0.3/0.5
**10**	9y 5 m	M	IC2	**+/+**	0.4/0.3/0.6	0.9/0.9/0.9
**11**	5y 4 m	M	IC2	**-/-**	0.2/0/0	0.5/0/0
**12**	7y 3 m	M	MHL2	**+/+**	1/0.6/0.4	0.4/0.3/0.3

### Primary outcome measures

The main goal of this study was to assess the acceptability of a serious game designed for GSE training in children with VH. The primary outcomes were the evaluation of undesirable side effects and children and parents’ acceptability of the video game. An adapted French version of The Child Simulator Sickness Questionnaire (SSQ) [22] was used to gather the children’s symptoms after each training session. The questionnaire contains seven questions evaluating the symptoms of simulator sickness, such as nausea, dizziness, headache, and eye discomfort, and is organized into three categories: Nausea, Oculomotor, and Disorientation (Figure S1A and S2B). Each question is answered with: “No,“ “A little,“ or “A lot.“ The score is assigned from 0 (no symptoms) to 2 (a lot of symptoms); a total score of 3 or more for any of the three symptom categories shows the presence of simulator sickness symptoms.

The Face Scale Pain-Revised (FPS-R) [23] was used to assess pain in the cervical region after each session. The FPS-R consists of six faces representing different facial expressions, from “no pain” to “most pain possible.“ The scale uses a 0 to 10 score, valid to assess pain in patients aged 4 to 12. The satisfaction of the children and their parents regarding the video game was evaluated at the end of the ten sessions using a list of 5 items presented orally (Figure S2A and S2B). A face Likert scale with 5 degrees of happiness was used for each item to assess children’s satisfaction levels. Parents’ satisfaction concerning the game was evaluated using a 5-point Likert scale, 1 being not satisfied at all and 5 being very satisfied.

### Secondary outcome measures

The secondary outcomes were the assessment of vestibular and motor function parameters. VOR gain was measured for all canals using the Video Head Impulse Test (VHIT, Synapsys®, Marseille, France), which uses a remote video camera detection to measure head and eye movements at high velocities (> 120–150°/s) [24]. VHIT evaluation had a twofold purpose: first, to determine a VOR deficit, defined in this study as a gain below 0.6 according to previous data [25, 26]; second, to evaluate VOR gain changes after the training. For VHIT evaluation, as of 5–6 years of age, children become more compliant; the procedure used is thus relatively similar to that used in adults [25].

DVA was assessed using a DVA commercialized system (Framiral®, Grasse, France). Static condition is measured by reading letters or symbol optotypes flashed for 50ms on a computer screen with the head stationary. Patients must move their head either laterally or vertically (right, left, up and down directions); the system was set to display optotypes while doing head rotations in the 150°/s to 300°/s range. Measures were done under both predictable and unpredictable conditions; patients moved their head actively (predictable condition) and the head of the patients was passively moved by one of the authors (unpredictable condition). Patients were allowed to watch each optotype up to three times in either and were asked to read the letter or symbol aloud. Corrective eyewear was used if necessary.

The Movement Assessment Battery for Children–Second Edition (MABC-2) was used to assess motor function. The MABC-2 is a test designed to evaluate and identify impairments in motor function performance in children from 3 to 16 years old [27]. This test contains eight items split into three categories: manual dexterity, ball skills, and static and dynamic balance. The test-retest values of the MABC-2 scale have been shown to represent a high level of reliability for the total score ICC = 0.83 [28].

### The serious game

**Fig. 1:**
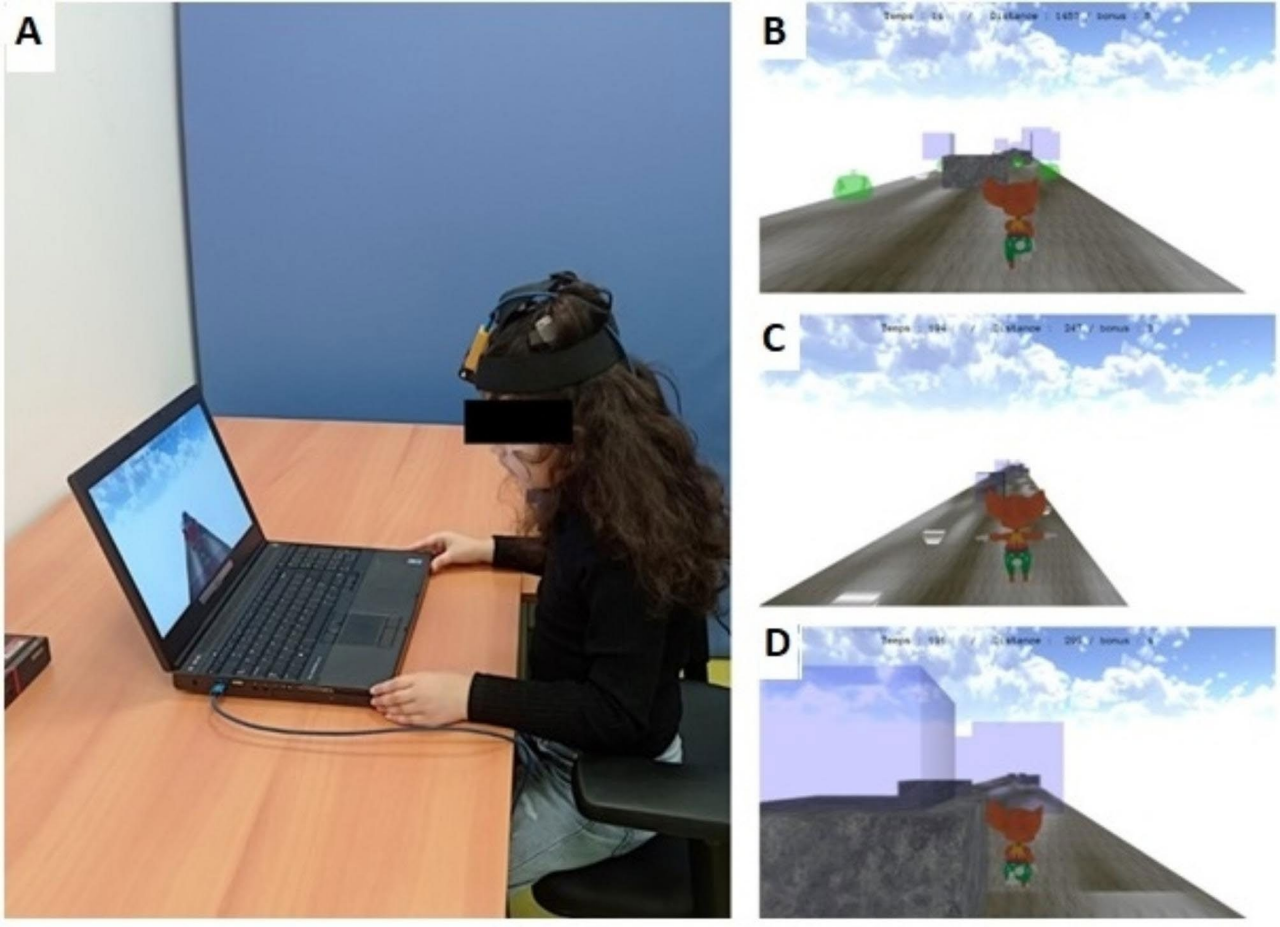
Serious game set up and visual presentation. (**A**) Set up of the serious game for each session. (**B,C,D**) screenshots of the game: the patient directs a moving character by turning the head rapidly up (jumping), down (crawling), left or right. Obstacles are to be avoided, rewards are to be collected

The KGR software was developed for this study; the video game was controlled and displayed on a classical laptop computer. The system used a head-mounted sensor to detect the angular speed of head movements in real-time (Fig. 1A). The patients wore a lightweight cap, and the sensor (MPU 6050) was placed on the center of the forehead. The sensor and accelerometers measured head accelerations along the horizontal, anterior, and posterior axes. The lightweight sensor contains a 3-axis accelerometer and a 3-axis gyroscope. The MPU data (acceleration and angle) were collected by the Arduino Nano, a programmable board containing a microcontroller and a USB port. The data were then transmitted to the software installed on the computer via the USB port in serial communication. The system allowed to determine a maximum and minimum angular speed threshold for each patient. The motion of the head triggers the task to be performed during the game in one or more directions only when the minimum head movement speed threshold is reached. For example, for a given patient, a minimum speed of 120°/s and a maximum speed of 250°/s can be determined: the rewarding interaction with the central character of the video game can only be triggered when the movement of the patient’s head reaches a speed of at least 120°/s; conversely, the game is paused when the speed of head movement reaches 250°/s.

The serious game scenario consisted of a 3D straight path suspended in the void along which different obstacles and items that give bonuses are randomly distributed (Fig. 1B). The playable character, the avatar, is represented by a mouse; the game’s objective is to collect as many items (teapots) as possible, avoiding obstacles, and without falling out of the path. The avatar is driven by head movements on the yaw and pitch plane; a head rotation to the right or the left drives the avatar to the right or the left, respectively. A head rotation upward makes the avatar jump over obstacles, and a head rotation downward makes the avatar crawl, allowing it to get through underneath obstacles. At the top of the screen is displayed the remaining time, the distance traveled, and the number of items collected.

### The procedure

Sessions were held at the hospital twice a week, at least 48 h apart, to avoid residual fatigue. All sessions were conducted in the presence of one of the three experimenters (JOS, PR, CB). The children underwent training with the serious game twice a week, for a total of ten sessions. During half of these sessions, once a week, they also underwent VR exercises modified for children’s abilities and motivational aspects. The classical VR approach consisted of 30-minute sessions conducted by a physiotherapist (JOS and CB), with a similar intervention approach to that described by Rine et al. in 2004 [29], which included eye-hand coordination, general coordination, and balance training. The level of difficulty of exercises was adapted to each subject’s ability, as is usually the case in clinical practice. The training with the serious game was split into four games of 2.5 min with a 1-minute rest between each game (total time: 15 min). After each session, side effects were assessed using the adapted French version of the Child SSQ (Figure S1 and S1bis) and the FPS-R visual analog scale for cervical pain evaluation.

### Pre- and post-evaluation

VHIT, active and passive DVA, and MABC-2 were performed before the training (T1) and five weeks later (T2). MABC-2 scores were collected at T1 and T2 by the same examiner. At the end of the training, both the children and parent 5-item satisfaction questionnaires were completed (Figure S2 and S2bis). In addition, the parents of the trained group were subjected to a semi-structured interview at the end of the training. They were asked, “What would you change about the game (general presentation, operation) or the procedure?“

### Statistical analysis

The statistical analyses were done using Statistica (v13 (2018), TIBCO Software Inc, Palo Alto, USA. http://tibco.com). Descriptive analyses were used to summarize the variables of interest. The non-parametric sign test was applied to compare baseline performance with the performance at the end of the training. A two-sided p-value (< 0.05) was set as statistically significant.

## Results

### Primary outcomes

All 12 children completed the ten sessions and received training as outlined in the procedure. Regarding the FPS-R evaluation, 8 children out of 12 (66%) reported discomfort during at least one of the sessions. This discomfort was transient as 3 out of 8 children did not report neck discomfort during another session, 4 out of 8 children had this discomfort during 2 sessions, and 1 child reported discomfort 3 times. In all cases, the discomfort was minimal and less than or equal to 3/10, except for one child who reported once a discomfort of 8/10, but 0/10 during the following session.

SSQ scores remained at 0 for nausea, ocular motricity, and disorientation at the end of each of the 10 sessions, except for one child who had a score of 5 and 10 for ocular motricity and disorientation, respectively, at the third session but who did not show discomfort afterward (Table 2).

**Table 2:** SSQ questionnaire scores in each section (nausea, ocular motricity, and disorientation) and children satisfaction questionnaire scores for each child. For SSQ, a score of 0 stands for an absence of side effects. For the satisfaction questionnaire, a score of 5 stands for the maximal level of satisfaction

	SSQ (mean scores for 10 sessions)			Children satisfaction questionnaire				
	Nausea (/3)	Occularmotricity (/3)	Disorientation (/3)	Clarity of the rules	Ease of use	Not tired after playing	Desire to play again	Game-related fun
**Child**								
1	0	0	0	5	5	5	5	5
2	0	0	0	5	5	5	5	5
3	0	0	0	5	5	3	5	5
4	0	0	0	5	5	5	5	5
5	0	0	0	5	5	5	5	5
6	0	0	0	5	5	4	3	5
7	0	0.5	1	5	5	5	5	5
8	0	0	0	5	5	5	5	5
9	0	0	0	5	5	5	5	5
10	0	0	0	5	5	5	5	5
11	0	0	0	5	5	4	3	5
12	0	0	0	5	5	5	5	5
**Mean**	0/3	0.04/3	0.08/3	5/5	5/5	4.67/5	4.67/5	5/5

Concerning the children’s satisfaction questionnaire at T2 (Table 2, Figure S2A), for questions 1, 2, and 5 (clarity of the rules, ease, and fun of the game), the children all rated 5/5 (very satisfied), resulting in an average satisfaction of 60/60. For question 3 (not tired after the game), the average satisfaction was 56/60; that is, 9 children rated it 5/5, 2 rated it a score of 4, and 1 rated it as 3. For question 4 (desire to play again), the average satisfaction was 56/60; that is, 10 out of 12 children rated with the maximum score of 5, whilst 2 rated the items with a score of 3.

Concerning the parent’s satisfaction questionnaire at T2 (Figure S2B), for questions 1, 2, and 4 (clarity of the rules, fun of the game, is the game well adapted to the child), parents all rated 5/5 (very satisfied), for an average satisfaction of 60/60. For question 5 (estimated benefit), the average satisfaction was 59/60. Question 3 was inverted (i.e., is the child tired after the game), and average satisfaction was 35/60.

Children also freely commented on the game, focusing mainly on the fun aspect of the game. Three children suggested choosing between several avatars, and two requested a higher difficulty level.

### Secondary outcomes

There was no significant difference in the VOR gain assessed by VHIT between T1 and T2 for the anterior, lateral, and posterior semicircular canals (p = 1, p = 0.55, and p = 0.75 on the right, and p = 0.77, p = 0.55 and p = 0.68 on the left, respectively).

**Fig. 2:**
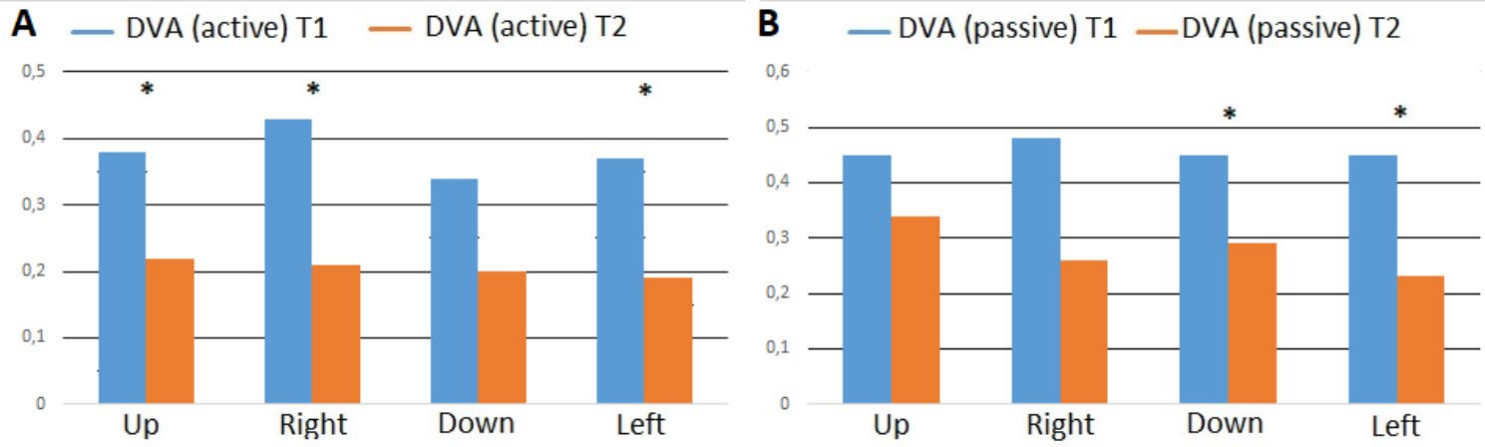
Mean active and passive dynamic visual acuity (DVA) scores in log Mar at T1 and T2 for up, right, down, and left directions

For active DVA, log Mar scores were significantly improved for the right, left, and up directions (0.19, 0.21, 0.22 at T2 versus 0.43, 0.47, 0.48 at T1, respectively, p < 0.05). Scores appeared to improve in the down direction, but not significantly (0.2 versus 0.34, p = 0.07; Fig. 2).

**Fig. 3:**
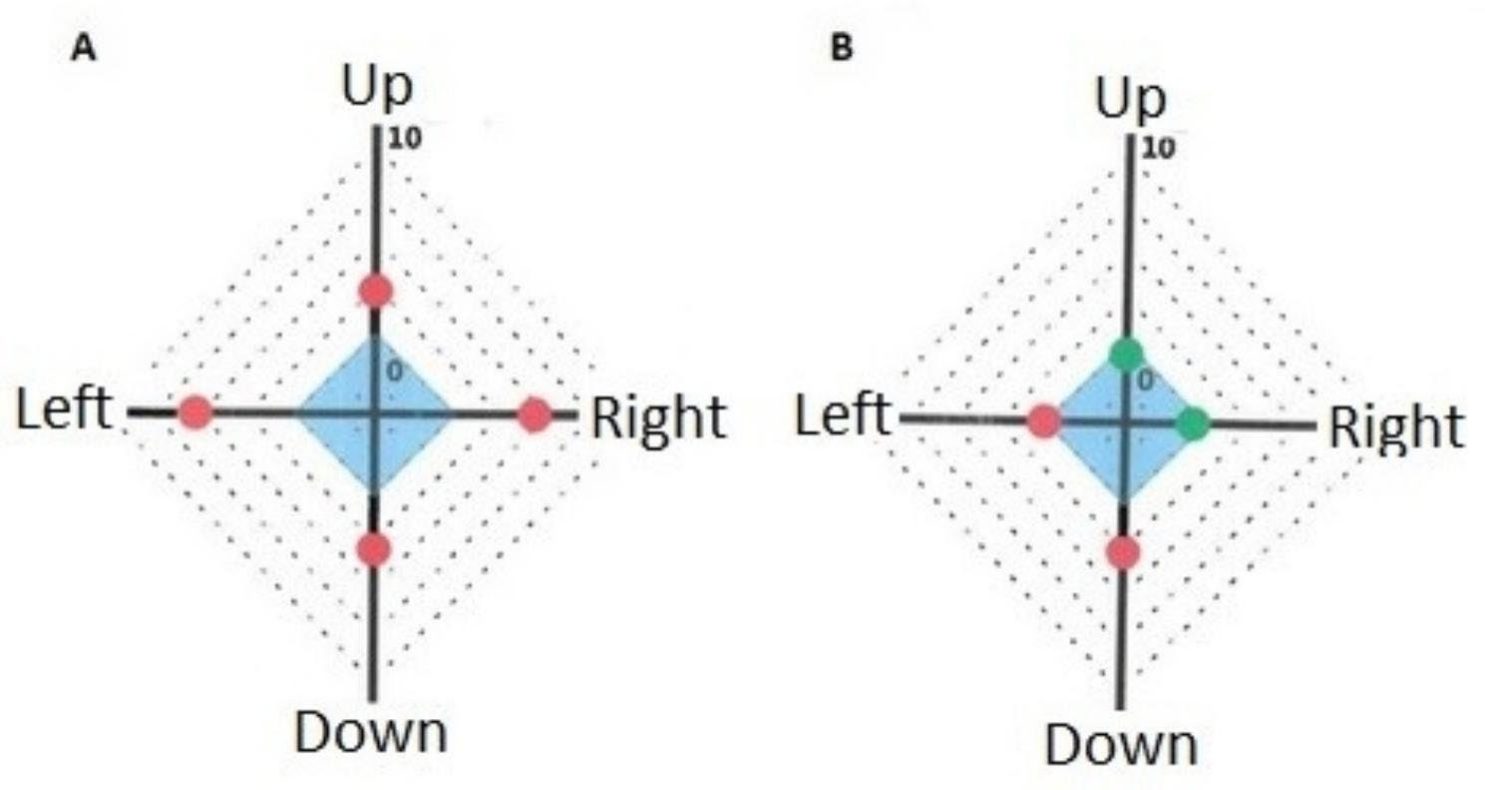
Active dynamic visual acuity of one of the participants at T1 (**A**), T2 (**B**). For each direction, the values are given from 0 (center) to 10 (end). Scores from 10 (low score) to 0 (high score) as given by the software before conversion into log MAR scores

The log Mar scores of passive DVA (Table 3) were significantly improved for the left and down directions (0.23 and 0.29 at T2 versus 0.45 and 0.45 at T1; p < 0.01 and p < 0.05, respectively). Scores improved for the right and up directions, but not significantly (0.26 and 0.34 at T2 versus 0.48 and 0.45 at T1; p = 0.08 and p = 0.75, respectively; Figs. 2 and 3).

**Table 3:** Dynamic Visual Acuity scores at T1 and T2 in log Mar

Child	T1 DVA (Active)	T1 DVA (Passive)	T2 DVA (Active)	T2 DVA (Passive)
	Down/Up/Left/Right	Down/Up/Left/Right	Down/Up/Left/Right	Down/Up/Left/Right
1	0.22/ 0.22/ 0.22/ 0.52	0.52/ 0.40/ 0.40/ 0.70	0.04/ 0.10/ 0/ 0.04	0/ 0.16/ 0.10/ 0.10
2	0.22/ 0.40/ 0.40/ 0.30	0.40/ 0.52/ 0.70/ 0.52	0.30/ 0.30/ 0.52/ 0.30	0.30/ 0.52/ 0.52/ 0.70
3	0.40/ 0.52/ 0.52/ 0.52	0.52/ 0.10/ 0.52/ 0.30	0.10/ 0.10/ 0/ 0.04	0.30/ 0.22/ 0.04/ 0.30
4	0.40/ 0.70/ 0.40/ 0.40	0.52/ 0.70/ 0.40/ 0.40	0.30/ 0.16/ 0.16/ 0.30	0.22/ 0.22/ 0.40/ 0.04
5	0.40/ 0.22/ 0.40/ 0.30	0.22/ 0.52/ 0.40/ 0.40	0.16/ 0.04/ 0.16/ 0.10	0.16/ 0.40/ 0.04/ 0.40
6	0.70/ 0.70/ 0.70/ 0.52	0.52/ 0.70/ 0.52/ 0.52	0.52/ 1/ 0.52/ 0.52	0.52/ 0.70/ 0.40/ 0.52
7	0.22/ 0.22/ 0.40/ 0.30	0.52/ 0.40/ 0.16/ 0.7	0.22/ 0.04/ 0.10/ 0.04	0.52/ 0.52/ 0.22/ 0.16
8	0.40/ 0.40/ 0.40/ 0.52	0.52/ 0.40/ 0.70/ 0.70	0.10/ 0.04/ 0.22/ 0.16	0.40/ 0.22/ 0.10/ 0.22
9	0.04/ 0.16/ 0.16/ 0.04	0.22/ 0/ 0.10/ 0.04	0.10/ 0.04/ 0.04/ 0.04	0.16/ 0.04/ 0.10/ 0.04
10	0.16/ 0.22/ 0.22/ 0.70	0.22/ 0.40/ 0.22/ 0.30	0.10/ 0.04/ 0.04/ 0.16	0.04/ 0.04/ 0.04/ 0.16
11	0.70/ 0.52/ 0.52/ 0.52	1/ 1/ 1 /1	0.40/ 0.70/ 0.52/ 0.70	0.70/ 0.70/ 0.70/ 0.52
12	0.22/ 0.30/ 0.16/ 0.52	0.22/ 0.22/ 0.30/ 0.22	0.04/ 0.04/ 0.04/ 0.10	0.16/ 0.30/ 0.10/ 0

**Fig. 4:**
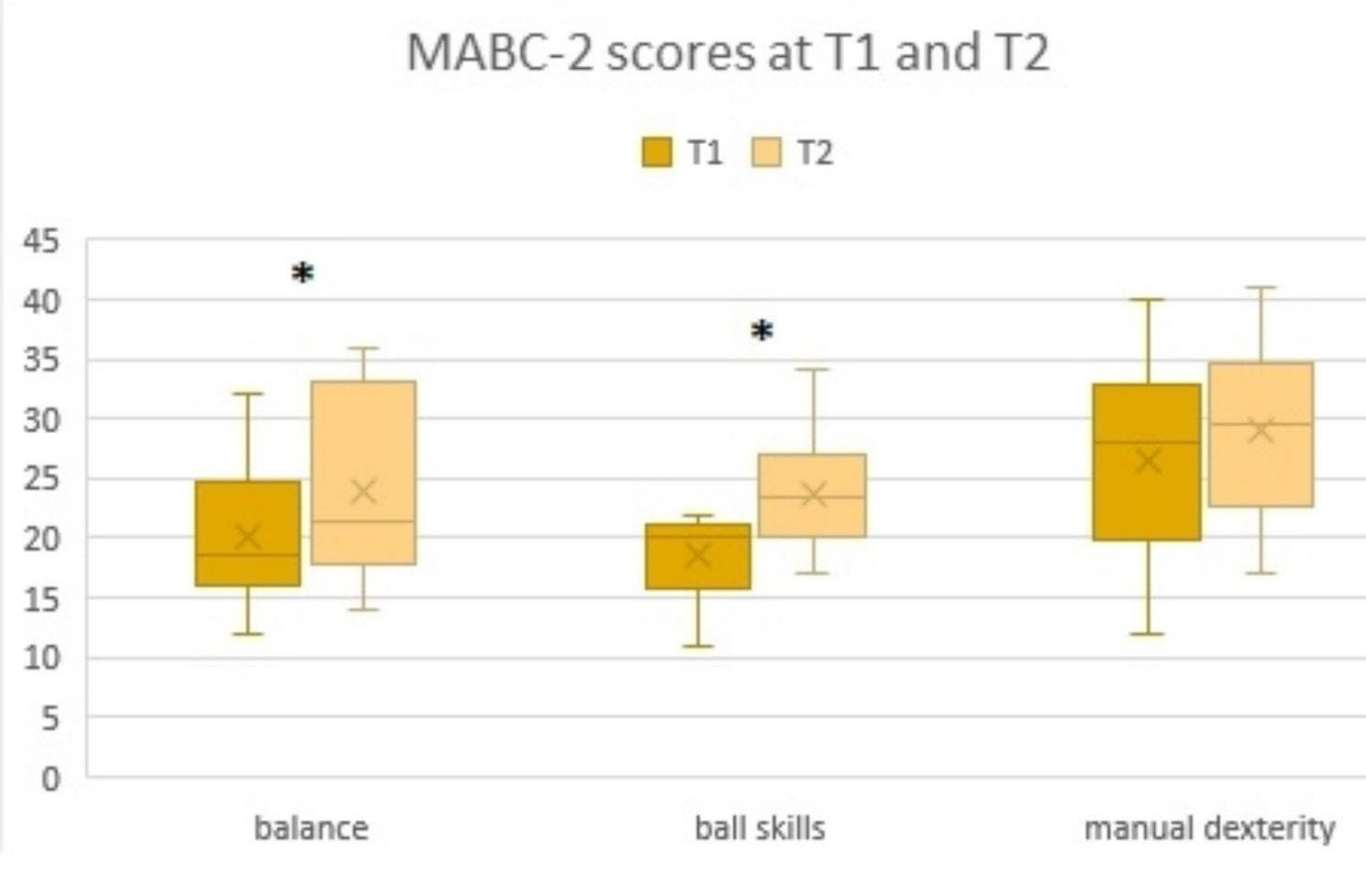
Mean MABC-2 scores before training (T1) and after training (T2) for each of the postural control section (balance, ball skills, and manual dexterity)

For the MABC-2 postural control scale (Table 4), the evaluation at T2 showed a significant improvement in the mean scores for the balance and ball skill sections compared to T1 (24 at T2 versus 20.1 at T1, p < 0.05 and 22.1 at T2 versus 18.6 at T1, p < 0.005, respectively; Fig. 4). There was no significant improvement in the manual dexterity section at T2 (31.8 versus 26.6, p = 0.15). For 4 children, the scores in the balance section were unchanged at T2; for the others, the scores were improved. For 1 child, the scores in the ball skill section were unchanged at T2; for the other 11, the scores were improved.

**Table 4:** MABC-2 scores at T1 and T2 for each of the three sections. MD: manual dexterity BS: Ball skills B: Balance

Child	T1 MD	T1 BS	T1 B	T2 MD	T2 BS	T2 B
1	12	20	18	17	27	20
2	22	18	15	19	19	14
3	30	11	19	28	20	33
4	25	19	16	26	34	22
5	33	15	16	34	20	16
6	32	22	20	35	24	20
7	17	21	12	22	30	21
8	30	14	17	32	17	17
9	19	20	26	25	23	36
10	40	21	29	39	27	33
11	26	22	21	31	24	22
12	33	20	32	41	20	34

## Discussion

This study tested the safety and efficacy of a 5-week rehabilitation program including a serious game dedicated to training gaze stability. Children with VH showed good tolerance and acceptability of the serious game KGR. After training with this rehabilitation system, their DVA significantly improved from the baseline assessment. In the future, interactive rehabilitation could be beneficial for children with VH. This approach may offer greater accessibility, delivery, and treatment compliance in young children with VH.

### Acceptability and feasibility outcomes

The duration of the game sessions did not exceed 15 min, and the 2 weekly sessions were separated by 48 h. For such a procedure, we noticed almost no ocular, visual, or nausea-type side effects nor spatial disorientation during each session. The rapid head movement during training (amplitude range 120–250°/sec) provoked little discomfort on the cervical spine. That is, just for one session only one child with a tension-type headache exhibited a high FPS-R score. Active head movement during training was chosen herein because voluntary active head movements are frequently used in daily life functional activities. Moreover, previous studies have shown that active GSE improve DVA scores during active (predictable) and passive (unpredictable) head rotations [30–32]. In addition, based on our experience, active head movements are better tolerated. All the sessions took place in the hospital under supervision (physiotherapist or physician) as it appears necessary to supervise the children during training to prevent non-adapted movements. As in adults, the child’s motivation could be essential for implementing VR using a serious game tool. Highly motivated individuals are more likely to engage in and persist longer at a particular activity [33]. Other criteria for adherence to training with a serious game include environment exploration, identification in an avatar, and rewards for progressing [33]. Considering such feasibility factors, the serious game applied herein was explicitly developed for this study. Although the software is a prototype with its content not being subject to validation in this study, we queried the implementation of this serious game. That is, children suggested several minor improvements, such as the adding of several avatars, the flexibility of adjusting the difficulty, or the option to track scores across sessions. A new version of the software, including a scoreboard, a choice between multiple avatars, multiple game settings (including multiple environments), and the auto-adaptive difficulty, could enhance the game’s adherence. These comments suggest that the children not only appreciated the game and were motivated, but equally suggest that the sessions were not perceived as a rehabilitation treatment. With such improvements, adherence could possibly be reinforced in young children. The relatively short duration of 5 weeks was chosen to ensure the patient’s participation. The high satisfaction rating at the end of the study furthermore indicated that the game training was well accepted. In this small sample of patients, no loss of sight was observed; all the children were able to finish the training, none was stopped prematurely and all 10 sessions (2 weekly sessions) were executed, even despite some geographical distance for some parents.

### Secondary outcomes

This clinical trial was not designed to compare this intervention with others.

However, being a new intervention, we aimed at determining the safety and efficacy of the serious.

game by measuring also secondary standard outcomes of vestibular rehabilitation.

Considering this, data showed a significant improvement in the DVA score in the active condition (predictable head movements) and, to a lesser extent, in the DVA passive condition assessment (unpredictable head movements). These results are in line with those reported by Herdman et al. in two randomized controlled trials, who found DVA improvement in passive but mainly active conditions, after GSE training in adults with VH [30, 31].

No significant difference in the VOR gain according to the VHIT assessment before and after the intervention was found herein. Previous studies indicate that positive changes in the DVA score are not necessarily correlated with VOR gain recovery [30, 31]. According to these studies, DVA improvement can be caused, at least in part, by changes in pre-programmed eye movements, like an increase in compensatory saccade frequency to compensate for VOR deficiency. Due to the primary objective of the present study, changes in compensatory saccades were not analyzed herein. One crucial issue is the duration of DVA improvement following the intervention. Therefore, future studies should assess the duration of DVA changes in the medium and long term.

Considering that the minimal detectable change of the MABC-2 score has been shown to be 5.76 [28], the results showed a significant improvement in postural control and eye-hand coordination abilities, as reported by the MABC-2 evaluation before and after the intervention. However, since no control group was included, it is difficult to distinguish whether the positive effects result from the combination of the serious game and classical physiotherapy intervention or are a consequence of the latter intervention alone. Indeed, the VR exercises used herein in combination with the serious game were similar to those used by Rine et al., who showed an improvement in motor skills in children with VH [29]. Further studies must assess the interest of combining serious games involving GSE and standard, customized VR intervention in the pediatric population with VH.

### Study limitations

Because study recruitment started during the Covid-19 pandemic, including more patients was challenging, and the ratio of male and female participants was uneven. The sample size and study design are somewhat limited in statistical power to have far-reaching conclusions regarding the secondary outcomes. Future studies need to include a larger number of participants and use a randomized control design to better understand the serious game’s efficacy on DVA patterns.

## Conclusion

The KGR serious game is a new system to complement VR in children with VH. The acceptability and feasibility of the game seem interesting according to the procedure used, at the frequency of 2 sessions of 15 min per week for five weeks, in combination with a weekly classical VR. Such a procedure might effectively improve DVA, ball skills, and balance. Although further studies are needed to confirm this, improved vestibular functioning may enhance the young child’s daily activities. This novel approach for young children with VH may assist their therapeutic effectiveness.

### Electronic supplementary material

Below is the link to the electronic supplementary material.

**S1**: Adapted French version of The Child Simulator Sickness Questionnaire (SSQ), called the Tolerance questionnaire for parents

**S1bis**: English translation of the adapted French version of The Child Simulator Sickness Questionnaire (SSQ)

**S2**:Children and parent satisfaction questionnaires. (A) Children satisfaction regarding the serious game was assessed using a 5-item Likert scale with 5 facial expressions. (B) Parent satisfaction was defined using another 5-item Likert with 5 numbers, 1 being not at all satisfied and 5 being very satisfied

**S2bis**: English translation of the Children’s satisfaction questionnaire (A) and Parent’s satisfaction questionnaire (B)

## Data Availability

The data presented in this study are available on request from the corresponding author. The data are not publicly available due to ethical, legal, and privacy issues.

## Abbreviations

cVEMPs: Cervical vestibular evoked myogenic potentials
DVA: Dynamic visual acuity
FPS-R: Face Scale Pain-Revised
GSE: Gaze stabilization exercises
KGR: Kid Gaze Rehab
Log Mar: Logarithm of the Minimum Angle of Resolution
MABC-2: Movement Assessment Battery for Children–Second Edition (MABC-2)
SSQ: Simulator Sickness Questionnaire
VH: Vestibular hypofunction
VHIT: Video Head Impulse Test
VOR: Vestibulo-ocular Reflex
VR: Vestibular Rehabilitation
