# Supplementary figures and images for: Developing a serious game for gaze stability rehabilitation in children with vestibular hypofunction
Source: J Neuroeng Rehabil. 2023 Sep 26;20:128. doi: 10.1186/s12984-023-01249-x (PMC10521575; doi:10.1186/s12984-023-01249-x)

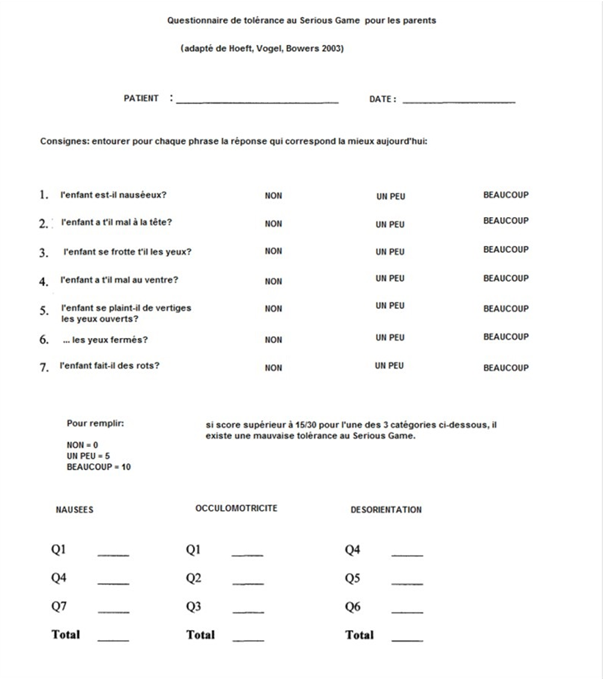

Supplement: Supplementary file 1 — S1: Adapted French version of The Child Simulator Sickness Questionnaire (SSQ), called the Tolerance questionnaire for parents [file 12984_2023_1249_MOESM1_ESM.bmp]

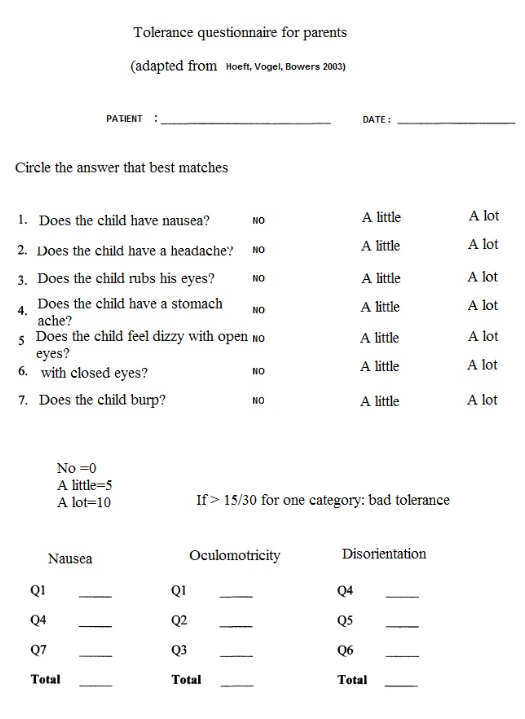

Supplement: Supplementary file 2 — S1bis: English translation of the adapted French version of The Child Simulator Sickness Questionnaire (SSQ) [file 12984_2023_1249_MOESM2_ESM.bmp]

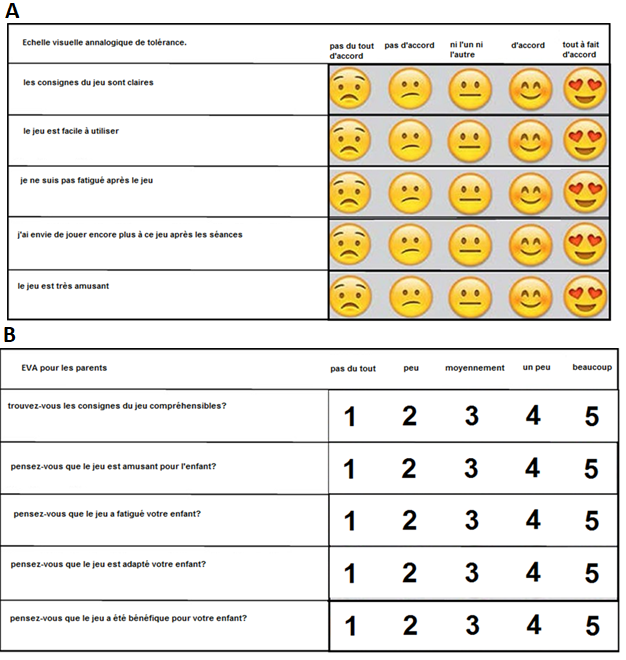

Supplement: Supplementary file 3 — S2:Children and parent satisfaction questionnaires. (A) Children satisfaction regarding the serious game was assessed using a 5-item Likert scale with 5 facial expressions. (B) Parent satisfaction was defined using another 5-item Likert with 5 numbers, 1 being not at all satisfied and 5 being very satisfied [file 12984_2023_1249_MOESM3_ESM.bmp]

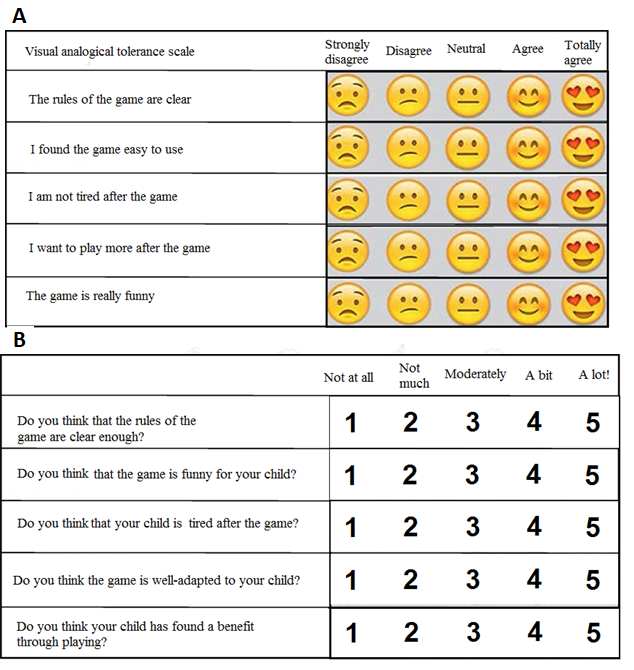

Supplement: Supplementary file 4 — S2bis: English translation of the Children’s satisfaction questionnaire (A) and Parent’s satisfaction questionnaire (B) [file 12984_2023_1249_MOESM4_ESM.bmp]
